# Short-term Outcomes of Polydioxanone Synthetic Mesh in 2-stage Breast Reconstruction: A Single-Center Comparative Study

**DOI:** 10.1093/asj/sjag019

**Published:** 2026-01-24

**Authors:** Erin N Abbott, Ruoying Li, Daniella King, Paulina E Chumakov, Elianna Dash, Carrie A Kubiak, Stephane A Braun, Allen Gabriel, Megan Vucovich, Lauren M Mioton, Galen Perdikis

## Abstract

**Background:**

Polydioxanone (PDO) mesh offers a more cost-effective option than biologic mesh for soft tissue support for 2-stage breast reconstruction with tissue expansion but differences in complication rates have not been well established.

**Objectives:**

The purpose of this study was to present the early postoperative complications recorded by a single center following immediate tissue expander (TE) insertion for breast reconstruction using PDO mesh, biologic mesh, or no mesh.

**Methods:**

A retrospective cohort study was performed of patients undergoing immediate TE insertion following mastectomy over a 3-year period (2021-2024) at a single academic center. Patients were grouped by mesh type. Univariate and multivariate regression analyses were performed.

**Results:**

A total of 919 TEs in 524 patients were analyzed: 27.7% with PDO mesh, 52.9% with biologic mesh, and 19.5% with no mesh. TE infection occurred in 70 breasts (7.6%) in 55 patients (10.5%), with infection rates of 10.6% in the PDO mesh group, 6.6% in the biologic mesh group, and 6.2% in the no mesh group (*P* = NS). Ipsilateral radiation exposure was the only factor significantly associated with infection (odds ratio, 7.2; *P* = .008). Prepectoral reconstructions resulted in higher infection rates than subpectoral cases, but mesh type was not independently associated with infection. Explantation occurred in 120 breasts (13.0%) with no difference between mesh types.

**Conclusions:**

Short-term outcomes following TE placement were comparable among patients receiving PDO mesh, biologic mesh, or no mesh. These findings suggest that mastectomy flap quality and patient factors may have a greater impact on complications than mesh type, especially with prepectoral placement.

**Level of Evidence: 3 (Therapeutic):**

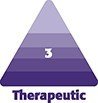

The use of mesh and other forms of internal soft tissue support matrices in 2-stage breast reconstruction has become increasingly common over the past 2 decades. These materials have been utilized both as a “sling” to provide increased inferolateral support for a tissue expander (TE) in subpectoral reconstruction and as “wraps” to provide additional soft tissue support and pocket stability in prepectoral reconstruction.^1-3^ First described for use in breast surgery in 2005, biologic meshes, such as acellular dermal matrices (ADMs), remain the most commonly used form of internal support.^4,5^ The use of biologic mesh in breast reconstruction has been associated with improved aesthetic outcomes, reduced capsular contracture rates, and potentially shorter time to second-stage reconstruction compared with reconstruction without biologic mesh.^6-10^

The use of biologic mesh in breast reconstruction can have drawbacks. Compared with TE reconstruction without mesh, early studies reported that biologic mesh resulted in higher rates of postoperative complications including seromas and surgical site infections.^5,6,10-14^ Although complication rates have improved with refinements in surgical technique and appropriate product utilization,^11^ cost remains a significant factor, with a single sheet of ADM often costing several thousand dollars.^15,16^ Given these concerns, a growing number of surgeons have turned to synthetic mesh as a lower-cost alternative.^17,18^ Both absorbable and nonabsorbable synthetic meshes have long been used in hernia and abdominal wall repair,^18,19^ but their application in breast surgery has been a relatively recent development. As such, outcomes data following synthetic mesh use in breast surgery remain an area for further study. The ability to draw broad conclusions about this category of materials is further complicated by the wide variability in design and biomechanical properties across different synthetic meshes.

A synthetic, monofilament polydioxanone (PDO) mesh, DuraSorb Monofilament Mesh (Integra LifeSciences, Princeton, NJ), was approved by the FDA in 2018 for soft tissue reinforcement. This material has a fine, flexible structure that fully resorbs in 9 to 12 months.^20,21^ Early reports are promising with low complication rates when used in both reconstructive and cosmetic breast surgery.^22,23^ However, these studies are limited by their small sample sizes. The purpose of this current study is to present a large single-center series focusing on early outcomes, particularly postoperative complications, with PDO mesh in 2-stage breast reconstruction. It was hypothesized that reconstruction with PDO mesh would be associated with a lower infection rate than reconstruction with biologic mesh.

## METHODS

### Study Population

This study was approved by the Vanderbilt University Medical Center (Nashville, TN) IRB (#241351). A retrospective chart review was conducted at a single tertiary care center with 6 surgeons performing breast reconstructions during the study period. Subjects were identified through departmental billing data. Patients who underwent first-stage immediate breast reconstruction with placement of TEs for reconstruction after mastectomy between July 1, 2021 and June 30, 2024 were included in the analysis. Analysis of second-stage reconstruction was not included in this study. Patients were excluded from this study if had undergone mastectomy or TE placement at an outside institution, had a previous TE placement, received TEs for non-oncoplastic indications, had delayed TE placement, or had received synthetic mesh other than PDO mesh. The biologic mesh products used during the study period were AlloDerm (LifeCell Corporation, Branchburg, NJ), SimpliDerm (Aziyo Biologics, Silver Spring, MD), and SurgiMend (Integra LifeSciences, Princeton, NJ).

### Patient Variables

Patient demographics, clinical data, and operative data were extracted from the electronic medical record. Age, BMI, diabetes diagnosis, smoking status, radiation history, and treatment with perioperative chemotherapy were recorded for each individual. Operative data included laterality, plane of reconstruction, mesh use, and mesh type. For the purposes of this study, TE placements with any degree of muscular coverage were classified as subpectoral. Patients were followed through March 20, 2025 for postoperative complications and TE removals. The primary outcome of interest was TE infection rate. Cases were identified as having an infection if there was documented clinical concern for infection. Signs and symptoms considered indicative of infection included erythema, purulent drainage, or aspiration of cloudy fluid. Microbiological confirmation was not a criterion for identifying cases of infection. Infections were included regardless of severity or management. Treatment of infections was left to the surgeon's discretion and could involve an outpatient course of antibiotics, inpatient admission for intravenous antibiotics, and/or operative intervention. Time to infection was documented where applicable. Secondary outcomes included other postoperative complications, namely, seroma of any volume, mastectomy flap necrosis, wound dehiscence, hematoma, TE device malfunction (including TE leak, rupture, and rotation), and expander explantation. Seroma was defined inclusively, with volumes as small as <5 mL counted if documented in postoperative notes, regardless of whether intervention was required. For patients who underwent explantation, the indication and time to explantation were recorded. Reinsertions and culture results were also documented where applicable.

### Surgical Technique

At the time of this study, TE placement had not yet been protocolized at this institution. As such, details regarding surgical technique were left to the attending surgeon's discretion. In general, however, the surgeons generally followed consistent institutional practices during the study period.

After completion of the mastectomy by the surgical oncology team, the reconstructive surgeon inspected the mastectomy flaps, with or without the assistance of intraoperative angiography with indocyanine green. In the absence of findings to suggest compromised blood flow to the mastectomy flap, the preferred approach was prepectoral TE placement. Tabbed TEs were then positioned and secured to the chest wall. Mesh configuration varied by plane of reconstruction but not mesh type. For TEs placed in the prepectoral plane, mesh was typically applied as either an anterior blanket or complete wrap. Mesh placed for subpectoral reconstructions was positioned as an inferolateral sling. The amount of mesh used was determined based on expander size and patient anatomy. In all cases, mesh was secured with 2-0 synthetic monofilament suture. Flap perfusion was rechecked after expander placement, and incisions were closed in multiple layers with absorbable suture.

### Statistical Analysis

Baseline demographics, operative data, complication rates, and TE explantation data were compared between mesh types. Statistical significance was defined a priori at an α of .05. The Kruskal-Wallis rank-sum test and either the chi-squared or Fisher's exact test were used to compare continuous and categorical variables, respectively. Post-hoc analyses were performed using pairwise Wilcoxon rank-sum tests for continuous variables and either pairwise Fisher's exact, chi-squared, or 2-proportion *z*-tests for categorical variables where appropriate. Bonferroni adjustments were applied to all post-hoc analyses.

Univariate regression modeling was performed to assess the relationship between preoperative and intraoperative risk factors and TE infections. A mixed-effects regression model was used to account for both patient- and breast-level data. The BMI term used in the model was a BMI-risk variable, which was defined as 0 for BMI <30 kg/m^2^ and increased linearly with BMI ≥30 kg/m^2^. Odds ratios (ORs), Wald confidence intervals, and *P*-values were computed from the fixed effects of the mixed-effects model. All statistical analyses were conducted using RStudio, v. 2024.12.1 (Posit PBC, Boston, MA).

## RESULTS

### Patient Demographics and Clinical Cohort

A total of 919 immediate TEs placed in 524 patients were included in the analysis. All patients included in this study were female. The median patient age was 51 years (range, 24-78 years). A total of 255 TEs (27.7%) in 145 patients (27.7%) were placed using PDO mesh, 487 TEs (52.9%) in 279 patients (53.2%) were placed using biologic mesh, and 177 TEs (19.5%) in 100 patients (19.1%) were placed without mesh. Within the biologic mesh group, Alloderm was used in 338 breasts (69.5%), SimpliDerm in 65 breasts (13.4%), and SurgiMend in 83 breasts (17.1%). There was no difference in age, smoking status, diabetes, preoperative or expansion phase radiation therapy, perioperative chemotherapy, or unilateral vs bilateral reconstruction between the groups. BMI was significantly lower in the group receiving biologic mesh compared with the PDO mesh (*P* = .024) or no mesh (*P* < .001) groups. The median time to follow-up among the entire cohort was 729.5 days (range, 268-1358 days) with significant differences between all 3 groups (*P* < .001; Table 1, post-hoc comparisons in Supplemental Table 1, available online at https://doi.org/10.1093/asj/sjag019).

**Table 1.** Patient-level and Breast-level Demographics and Clinical Characteristics^a^

Patient-level data	PDO mesh *n* = 145	Biologic mesh *n* = 279	No mesh *n* = 100	*P*-value
Age (years)	50.0 [29-74]	50.0 [24-77]	51.5 [24-78]	.551
BMI (kg/m^2^)	28.3 [18.8-47.9]	26.8 [17.9-44.6]	30.0 [17.8-51.2]	<.001
Current smoker	6 (4.1%)	16 (5.7%)	9 (9.0%)	.280
Diabetes	20 (13.8%)	21 (7.5%)	14 (14.0%)	.061
Perioperative chemotherapy	51 (35.2%)	86 (30.8%)	34 (34.0%)	.630
Bilateral reconstruction	113 (77.9%)	206 (73.8%)	75 (75.0%)	.693
Time to follow-up (days)	499 [269-820]	923 [268-1358]	765 [276-1352]	<.001
Breast-level data				
Ipsilateral radiation	23 (9.0%)	54 (11.1%)	14 (8.0%)	.414
Prepectoral TE placement	218 (85.5%)	316 (65.0%)	142 (80.7%)	<.001

Values are median [range] or n (%). PDO, polydioxanone; TE, tissue expander. ^a^All patients included in the cohort were female.

Overall, 677 TEs (73.7%) were inserted in the prepectoral plane and 242 TEs (26.3%) were inserted in the subpectoral plane. A greater percentage of TEs were prepectoral in the PDO mesh group compared with the biologic (*P* < .001) and no mesh (*P* = .001) groups.

**Figure 1.**
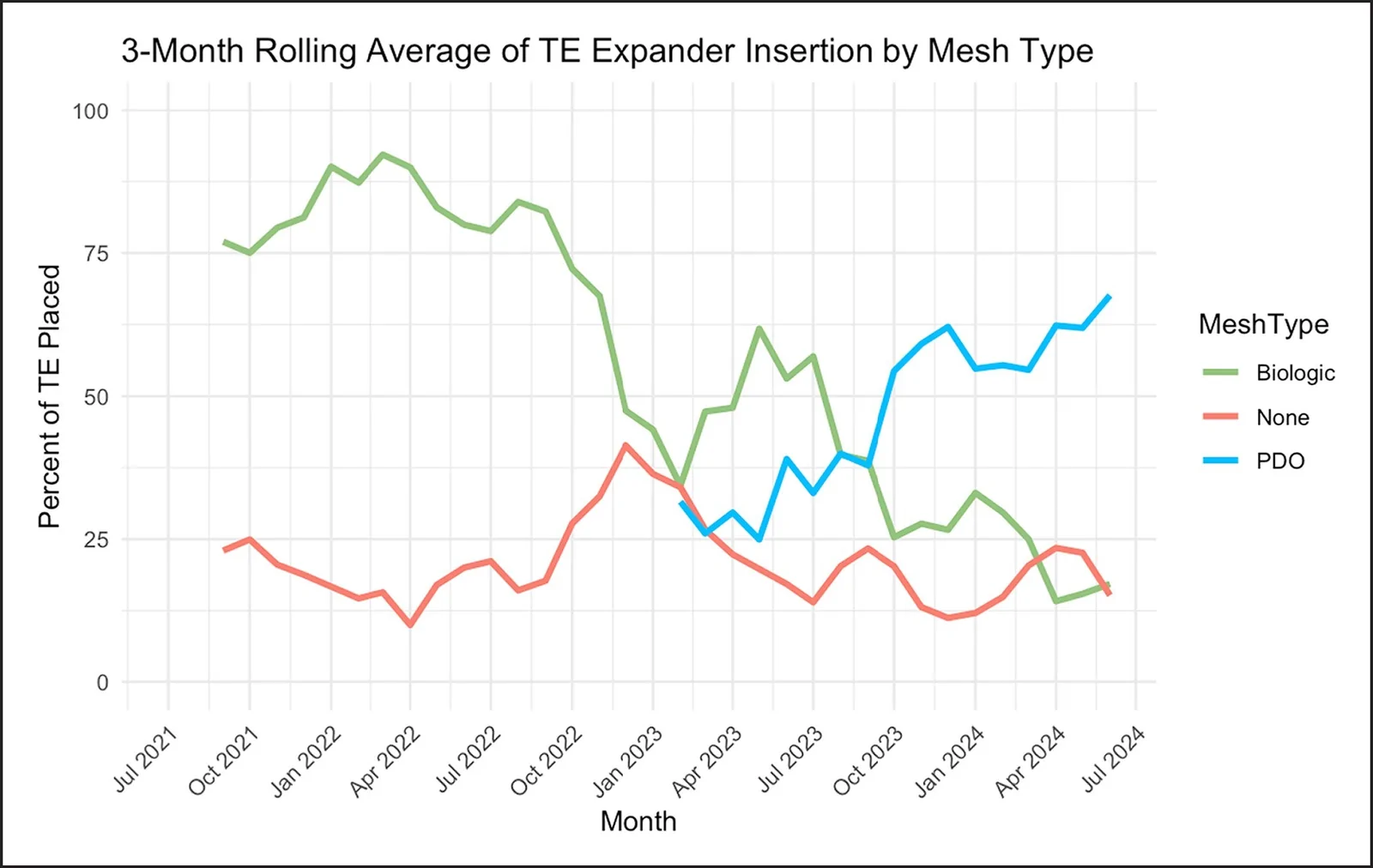
Three-month rolling average of tissue expander insertion by mesh type. Three-month rolling average of mesh utilization over time among immediate TE placements. The proportion of TEs placed using biologic mesh (green), PDO mesh (blue), and no mesh (red) is plotted for the study period. PDO, polydioxanone; TE, tissue expander.

Rates of mesh utilization varied throughout the study period. At the beginning of the study, approximately 75% of all TEs were placed using biologic mesh and 25% were placed without mesh. With the introduction of PDO mesh into clinical practice in January 2023, the utilization rate for PDO mesh surpassed that of biologic mesh in August 2023. By the end of the study period, 71% of TEs were placed using PDO mesh, 19% using biologic mesh, and 10% without mesh (Figure 1).

### Tissue Expansion Complications: All Patients

In total, 55 patients (10.5%) with 70 breast reconstructions (7.6%) developed a TE infection. Of the infected TEs, 27 (10.6%) were placed with PDO mesh, 32 (6.6%) were placed with biologic mesh, and 11 (6.2%) were placed with no mesh. There was no significant difference in infection rates between the groups. The median time to infection was not significantly different between groups. Rates of wound dehiscence were higher in breasts with PDO mesh than in breasts without mesh (*P* = .023). Seroma formation rates were significantly different across all groups (*P* = .025); however, these differences were no longer statistically significant after Bonferroni correction in the post-hoc analyses. There were no significant differences between flap necrosis, hematoma, and TE device malfunction rates between groups (Table 2; post-hoc analyses in Supplemental Table 2, available online at https://doi.org/10.1093/asj/sjag019). Radiation therapy prior to or during the expansion phase (*P* = .008; OR, 7.215; 95% CI, 1.672-31.134) was the only variable significantly associated with infection on univariate regression analysis (Supplemental Table 3, available online at https://doi.org/10.1093/asj/sjag019).

**Table 2.** Breast-level Complications Among All TEs

	PDO mesh *n* = 255	Biologic mesh *n* = 486	No mesh *n* = 176	*P*-value
Surgical site infection	27 (10.6%)	32 (6.6%)	11 (6.3%)	.111
Time to infection (days)	32 [7-176]	42 [4-232]	46 [11-127]	.728
Seroma	87 (34.1%)	128 (26.3%)	62 (35.2%)	.025
Flap necrosis	29 (11.4%)	37 (7.6%)	15 (8.5%)	.228
Wound dehiscence	19 (7.5%)	19 (3.9%)	3 (1.7%)	.012^a^
Hematoma	5 (2.0%)	13 (2.7%)	4 (2.3%)	.919
TE device malfunction	6 (2.4%)	6 (1.2%)	3 (1.7%)	.505

Values are median [range] or n (%). PDO, polydioxanone; TE, tissue expander.

### Tissue Expander Complications: By Plane of Reconstruction

Because the plane of reconstruction may influence the decision to use mesh, complication rates were first compared between subpectoral and prepectoral TEs to isolate the impact of mesh type from plane of reconstruction. Compared with the subpectoral group, the prepectoral group had a higher median BMI (*P* < .001) and a higher percentage of patients receiving chemotherapy (*P* = .044). The percentage of current smokers was greater in the subpectoral group (*P* = .008; Supplemental Table 4, available online at https://doi.org/10.1093/asj/sjag019). Compared with subpectoral TE placement, prepectoral TEs were associated with higher overall rates of infection (*P* = .012) and seroma (*P* < .001). There was no difference in flap necrosis, wound dehiscence, hematoma, or TE complication between the pre- and subpectoral groups (Table 3).

**Table 3.** Breast-level Complications by Plane of Reconstruction

	Prepectoral *n* = 676	Subpectoral *n* = 241	*P*-value
Surgical site infection	61 (9.0%)	9 (3.7%)	.012
Time to infection (days)	35 [4-232]	45 [9-176]	.593
Seroma	236 (34.9%)	41 (17.0%)	<.001
Flap necrosis	58 (8.6%)	23 (9.5%)	.749
Wound dehiscence	34 (5.0%)	7 (2.9%)	.234
Hematoma	15 (2.2%)	7 (2.9%)	.725
TE device malfunction	14 (2.1%)	1 (0.4%)	.134

Values are median [range] or n (%). TE, tissue expander.

Among the subgroup of prepectoral TEs, the BMI was significantly higher in patients with no mesh compared with the biologic mesh group (*P* = .003; Supplemental Table 5, available online at https://doi.org/10.1093/asj/sjag019, post-hoc analysis in Supplemental Table 6, available online at https://doi.org/10.1093/asj/sjag019). Although rates of wound dehiscence (*P* = .013) were higher among breasts with PDO mesh compared with breasts with no mesh, overall infection rates were not statistically different between groups (Table 4; post-hoc analyses in Supplemental Table 7, available online at https://doi.org/10.1093/asj/sjag019). Radiation therapy prior to or during expansion (*P* = .044; OR, 5.136; 95% CI, 1.044-25.277) was the only variable significantly associated with TE infection among prepectoral reconstructions on univariate regression analysis. No other variables, including mesh type, were significantly associated with TE infection on univariate analysis (Supplemental Table 8, available online at https://doi.org/10.1093/asj/sjag019).

**Table 4.** Breast-level Complications Among Prepectoral Tissue Expanders

	PDO mesh *n* = 218	Biologic mesh *n* = 316	No mesh *n* = 142	*P*-value
Surgical site infection	25 (11.5%)	26 (8.2%)	10 (7.0%)	.285
Time to infection (days)	28.0 [7-103]	44.0 [4-232]	26.5 [11-127]	.378
Seroma	79 (36.2%)	104 (32.9%)	53 (37.3%)	.580
Flap necrosis	25 (11.5%)	23 (7.3%)	10 (7.0%)	.180
Wound dehiscence	18 (8.3%)	14 (4.4%)	2 (1.4%)	.012
Hematoma	5 (2.3%)	7 (2.2%)	3 (2.1%)	1.000
TE complication (leak, rupture, rotation)	6 (2.8%)	5 (1.6%)	3 (2.1%)	.644

Values are median [range] or n (%). PDO, polydioxanone; TE, tissue expander.

### Tissue Expander Explantation

A total of 120 TEs (13.0%) in 87 patients (16.4%) were explanted prior to final reconstruction. The most common indications for explant were TE infection (n = 54; 45.0% of all explanted TE), exposed TE or threatened exposure (n = 28, 23.3% of explanted TE), and no further desire to pursue reconstruction (n = 12, 10.0% of explanted TE). There were no differences in overall explant rate between groups (Table 5). Culture results from explants were similar between mesh types (Supplemental Table 9, available online at https://doi.org/10.1093/asj/sjag019).

**Table 5.** Breast-level Indication and Timing of Tissue Expander Removals

	PDO mesh *n* = 255	Biologic mesh *n* = 486	No mesh *n* = 176	*P*-value
Number of explants	38 (14.9%)	57 (11.7%)	25 (14.2%)	.423
Explant due to complication	35 (13.7%)	47 (9.7%)	18 (10.2%)	.231
Infection requiring TE removal	19 (7.5%)	25 (5.1%)	10 (5.7%)	.444
Threatened or visible TE exposure	10 (3.9%)	15 (3.1%)	3 (1.7%)	.421
Concern for spread of contralateral infection	1 (0.4%)	1 (0.2%)	1 (0.6%)	.765
Chronic seroma	2 (0.8%)	2 (0.4%)	1 (0.6%)	.840
Extensive flap necrosis	1 (0.4%)	1 (0.2%)	1 (0.6%)	.765
TE complication (leak, rupture, rotation)	2 (0.8%)	3 (0.6%)	2 (1.1%)	.782
Time to explant (days)	51.5 [9-367]	67 [10-882]	64 [9-309]	.253
TE reinserted^a^	7 (18.4%)	18 (31.6%)	4 (16.0%)	.192

Values are median [range] or n (%). PDO, polydioxanone; TE, tissue expander. ^a^Percentage of explanted TEs.

## DISCUSSION

In this single-institution study, there were no significant differences in overall TE infection or explant rate among patients undergoing 2-stage breast reconstruction with PDO mesh, biologic mesh, or no mesh. The only predictor for TE infection identified in the cohort was ipsilateral exposure to radiation therapy. These findings suggest mesh use and mesh type may make a more limited contribution to TE infections than other clinical factors such as mastectomy flap quality and radiation.

Biologic meshes, such as ADM, have been the predominant adjunct used for soft tissue support in breast surgery for the past 2 decades. In the United States, the overall rate of biologic mesh use has continued to show steady growth into the early 2020s.^12^ However, increasing concerns regarding healthcare costs and continued advances in tissue engineering have led to a shift towards the use of synthetic mesh for soft tissue support. As the assortment of options for soft tissue support continues to expand, changes in surgical practice are expected to follow. PDO mesh was the predominant product at this institution during the study period and has gained increasing interest nationally since FDA approval in 2018 due to its lower cost, favorable handling characteristics, and predictable resorption profile. Institutional data demonstrate a steady increase in the use of PDO mesh use since its introduction in 2023. As surgical practice continues to evolve, developing an evidence-based understanding of the impact of new technologies on patient outcomes remains important.

Previous studies examining PDO mesh use in the context of aesthetic and revision breast surgery suggest favorable safety and aesthetic outcomes.^13-15^ In over 18 months of PDO mesh use for immediate TE insertion at this institution, 10.6% of breasts with PDO mesh developed an infection, and 7.5% required explantation for infection source control. These rates were nominally higher but not statistically different compared with infection or explant rates in the biologic and no mesh groups. This is largely consistent with existing literature on experiences with PDO mesh for breast reconstruction. In a small series of 14 prepectoral breast reconstructions, Qiu and Seth report 1 infection (7.1%) requiring expander removal.^16^ Taken together, the early experience data suggest that short-term outcomes with PDO mesh are comparable to those with biologic mesh or no mesh. These findings are consistent with previous work showing similar or lower rates of infection, and reoperation between other synthetic meshes and biologic meshes.^17-19,24^ Similar findings have suggested no significant differences in complication rates between synthetic mesh–assisted reconstructions and reconstructions without mesh.^20,21,25-27^ However, further research regarding long-term outcomes, especially with regards to pocket stability, is warranted. Compared with biologic mesh, PDO mesh is a stiffer material that provides greater initial rigidity but less long-term support after resorption.^22^ By comparison, biologic mesh offers greater conformability and stretch during expansion. These material differences may influence tissue dynamics and long-term pocket stability, even if early complication rates appear similar.

Interestingly, a higher rate of Gram-negative positive cultures was observed in the PDO mesh group. This finding, although not statistically significant, may reflect patient selection factors, some of which may be unaccounted for (eg, BMI, volume of breast tissue resected) as well as potential differences in the interaction between bacterial species and mesh type. Breast surgery is typically considered a clean operation, and the expected organisms are usually Gram-positive skin flora. In the case of Gram-negative infections, Klein et al discuss 4 possible sources of infection originally proposed by Nahabedian et al.^23,28^ Klein et al suggest that the most likely sources of Gram-negative infection include seeding from transient bacteremia from other medical interventions or overgrowth of Gram-negative species secondary to use of perioperative antibiotics aimed at covering Gram-positive organisms. There are some data to suggest that differences in material properties of mesh may influence bacterial adhesion and even the inflammatory response to bacterial infection.^29,30^ Given these data, it would not be unreasonable to expect that the material properties of PDO mesh may predispose the adjunct to Gram-negative colonization, although this remains speculative and warrants further study.

This study also evaluated the relationship between mesh use or type and the development of mediating complications that have previously been shown to increase the likelihood of developing a TE infection (eg, wound dehiscence, mastectomy flap necrosis, and seroma).^31-34^ In both the main analyses and subgroup analyses, rates of wound dehiscence were higher among TEs with PDO mesh than for TEs with no mesh. However, there were no differences in rates of wound dehiscence between PDO mesh and biologic mesh. Currently, there is no consensus on the relationship between mesh type/use and rates of wound dehiscence. Some studies show no difference in rates of wound dehiscence between synthetic mesh and no mesh reconstructions.^25,35^ However, the evidence comparing biologic mesh to synthetic mesh has been mixed. Some studies have suggested higher rates of wound dehiscence with biologic mesh, while others have found no difference.^36-38^ Comparison of wound dehiscence rates and wound healing complications between mesh types should be interpreted with some caution because the unique properties of each individual mesh product, as well as the placement of the mesh, may impact the amount of tension placed on the closure. Although not measured in this study, PDO mesh was subjectively found to be easier to handle and smooth out than biologic mesh. Given the lack of difference in wound dehiscence between biologic and no mesh groups, the observed increase in wound dehiscence with PDO mesh compared with no mesh may be related to other underlying variables not captured in this study. These factors could include initial TE fill, incision design, volume of breast tissue resected, or the amount of tension across the mastectomy flap causing decreased blood flow.

While there were no other associations between mesh type and other mediating complications, a significantly higher rate of seroma formation was found among prepectoral TEs compared with subpectoral TEs. Other studies have produced this same finding,^39,40^ and, taken together, these data support the role of poor mastectomy flap vascularity in seroma formation. Hypovascularity contributes to proinflammatory conditions, diminished lymphatic flow, and capillary leakage.^31,39,41^ This assertion is further supported by higher rates of seromas in patients with larger mastectomy resections where there is a higher likelihood of ischemic injury to the overlying soft tissue.^31,39,41^ Some previous studies have suggested that mesh, particularly biologic mesh, may also contribute to seroma formation.^42-44^ Seroma rates with synthetic mesh may be lower or equivalent to seroma rates with biologic mesh, and there are some data to suggest that seroma rates with synthetic mesh are not significantly different than seroma rates without mesh.^17-19,24,37^ No difference in seroma rates was found between mesh groups after controlling for plane of reconstruction, which suggests the type of internal soft tissue support does not impact seroma rate. It is also important to note that the absolute seroma rates appear higher than in some reports, which may be attributed to the broadly inclusive definition for postoperative seroma or potential variations in drain placement and removal practices.

This study did not identify any difference in explant rates between mesh types. Mesh use and choice of mesh may not have a significant impact on short-term outcomes and a patient's ability to progress through their reconstructive journey. Rather, as demonstrated by the singular significance of radiation on regression analysis and a significantly higher infection rate among prepectoral TEs, the findings suggest there may be a greater effect from the quality of the overlying soft tissue on the development of TE complications. The importance of a well-vascularized mastectomy flap is illustrated in a recent report by Bekisz et al. In their cohort of 826 cases, they found an increased incidence of isolated wound dehiscence in breasts with prepectoral TEs compared with breasts with total submuscular TEs. From these results, they concluded that the absence of an intervening well-vascularized soft tissue layer decreases skin tolerance for postoperative insults.^45^ However, this is not to say surgeons should not offer prepectoral reconstruction. Prepectoral reconstructions offer a number of advantages over subpectoral placement, including decreased chest wall morbidity, animation deformities, early postoperative pain, and improved patient-reported outcomes.^46-55^ In patients with well-vascularized mastectomy flaps, prepectoral reconstruction may still be a suitable option, although further research is needed to define the appropriate patient population for this approach and to explore for any potential interactive effects between choice of soft tissue support and plane of reconstruction.

This study reports on the largest cohort of PDO mesh patients to date and is the first to provide a direct comparison of short-term outcomes and complications among immediate TE placements using PDO mesh, biologic mesh, and no mesh following mastectomy. Nonetheless, several limitations should be considered. The retrospective design and inclusion of multiple surgeons, while reflective of real-world practices, limited the ability to control factors relating to mesh selection, surgical decision-making, and operative technique. The study also spanned a 3-year period during which institutional PDO mesh use increased substantially. Initially, PDO mesh use was reserved for patients with healthy mastectomy flaps undergoing prepectoral reconstruction. However, with time and increased familiarity with the product, PDO mesh use grew. These changes in practice have introduced selection bias into the study. The inclusion of multiple surgeons increased the sample size and improved the generalizability of the findings, but also introduced potential confounding variables because variations in surgical judgement and preferences could not be controlled. Additionally, factors that have influenced surgical decision-making, such as mastectomy flap thickness and intraoperative angiography findings, were not routinely recorded and therefore may have limited the ability to control selection bias.

Infection rates may have also been influenced by the inherent limitations of retrospective review. Because the definition for an infection was based on clinical diagnosis, it relied in retrospective discernment of true infection from inflammatory mimics. Additionally, minor infections that resolved with oral antibiotics between clinic visits may not have been fully captured if not documented. This study captures infections that occurred before and after initiating expansions. Accessing the device to perform an expansion, even if done under sterile conditions, introduces risk for infection and confounds the ability to make stronger conclusions about the contribution of various factors to TE infection. For these reasons, caution should be used when extrapolating the findings to mesh use in the setting of direct-to-implant reconstruction. The ability to draw conclusions, particularly within subgroup analyses, was further limited by a relatively small absolute number of infections. This challenge was compounded by the presence of multiple comparison groups, increasing the risk of Type II error.

Finally, bioabsorbable meshes may demonstrate 2 peaks of complications: one in the early postoperative period and a second after resorption at approximately 1 year. This study focused on complication rates during the expansion phase and therefore may not have captured this second peak in complications. With longer term follow-up of this cohort, future studies may examine long-term outcomes and rates of revision surgery based on patient and surgical factors at the time of TE placement.

## CONCLUSIONS

This study presents the largest known cohort examining short-term outcomes of PDO mesh in 2-stage breast reconstruction. Overall complication rates, including infection and explantation, were comparable among reconstructions using PDO mesh, biologic mesh, and no mesh. Although rates of seroma and wound dehiscence varied by group, these findings were not consistent across subgroups or regression analysis, suggesting a multifactorial etiology. Further research is needed to assess long-term outcomes and define the optimal patient population for PDO mesh use. Until such work can be completed, mesh selection may be reasonably guided by other factors, such as cost, patient autonomy, and surgeon preference.

## Supplemental Material

This article contains supplemental material located online at https://doi.org/10.1093/asj/sjag019.

## Supplementary Material

sjag019_Supplementary_Data
