# Supplementary material for: Short-term Outcomes of Polydioxanone Synthetic Mesh in 2-stage Breast Reconstruction: A Single-Center Comparative Study
Source: Aesthet Surg J. 2026 Jan 24;46(10):1101–8. doi: 10.1093/asj/sjag019 (PMC13577730; doi:10.1093/asj/sjag019)
Supplement: Supplementary file 1 [file sjag019_supplementary_data.zip › Supplemental Table 1.docx]

**Supplemental Table 1.** Post-hoc Comparisons of Significant Demographic and Clinical Characteristics from Table 1^1^

| Variable | Comparison groups (measure) | p-value | Adjusted p-value^2^ |
| --- | --- | --- | --- |
| BMI (median) | Biologic (26.7) vs. PDO mesh (28.3) | 0.008 | 0.024 |
|  | Biologic (26.7) vs. None (30.0) | <0.001 | <0.001 |
|  | PDO mesh (28.3) vs. None (30.0) | 0.069 | 0.208 |
| Time to follow-up (median) | Biologic (923) vs. PDO mesh (499) | <0.001 | <0.001 |
|  | Biologic (923) vs. None (765) | <0.001 | 0.001 |
|  | PDO mesh (499) vs. None (765) | <0.001 | <0.001 |
| Prepectoral TE placement (%) | Biologic (65.0%) vs. PDO mesh (85.5%) | <0.001 | <0.001 |
|  | Biologic (65.0%) vs. None (80.7%) | <0.001 | 0.001 |
|  | PDO mesh (85.5%) vs. None (80.7%) | 0.234 | 0.701 |

^1^Pairwise two-proportion z-test used. ^2^Bonferroni adjustment applied.
