# Supplementary material for: Short-term Outcomes of Polydioxanone Synthetic Mesh in 2-stage Breast Reconstruction: A Single-Center Comparative Study
Source: Aesthet Surg J. 2026 Jan 24;46(10):1101–8. doi: 10.1093/asj/sjag019 (PMC13577730; doi:10.1093/asj/sjag019)
Supplement: Supplementary file 1 [file sjag019_supplementary_data.zip › Supplemental Table 2.docx]

**Supplemental Table 2**. Post-hoc Comparisons of Breast-Level Complications from Table 2^1^

| Variable | Comparison groups (%) | p-value | Adjusted p-value^2^ |
| --- | --- | --- | --- |
| Seroma (%) | Biologic (26.3%) vs. PDO mesh (34.1%) | 0.027 | 0.080 |
|  | Biologic (26.3%) vs. None (35.4%) | 0.026 | 0.077 |
|  | PDO mesh (34.1%) vs. None (35.4%) | 0.812 | 1.000 |
| Wound dehiscence (%) | Biologic (3.9%) vs. PDO mesh (7.5%) | 0.038 | 0.114 |
|  | Biologic (3.9%) vs. None (1.7%) | 0.162 | 0.486 |
|  | PDO mesh (7.5%) vs. None (1.7%) | 0.008 | 0.023 |

^1^Pairwise Fisher’s exact and Chi-squared tests used. ^2^Bonferroni adjustment applied.
