# Supplementary material for: Short-term Outcomes of Polydioxanone Synthetic Mesh in 2-stage Breast Reconstruction: A Single-Center Comparative Study
Source: Aesthet Surg J. 2026 Jan 24;46(10):1101–8. doi: 10.1093/asj/sjag019 (PMC13577730; doi:10.1093/asj/sjag019)
Supplement: Supplementary file 1 [file sjag019_supplementary_data.zip › Supplemental Table 3.docx]

**Supplemental Table 3**. Univariate Regression Analysis of Pre- and Intraoperative Risk Factors for TE Infection

|  | p-value | OR [95% CI] |
| --- | --- | --- |
| Age | 0.806 | 1.009 [0.942 – 1.080] |
| BMI | 0.606 | 1.055 [0.860 – 1.296] |
| Ipsilateral radiation therapy | 0.008 | 7.198 [1.669 – 31.051] |
| Prepectoral plane of reconstruction | 0.394 | 2.564 [0.295 – 22.322] |
| Perioperative chemotherapy | 0.707 | 1.354 [0.278 – 6.600] |
| Diabetes | 0.897 | 1.174 [0.104 – 13.230] |
| Current smoker | 0.888 | 1.232 [0.068 – 22.379] |
| PDO mesh | 0.474 | 1.859 [0.340 – 10.157] |
| No mesh | 0.967 | 1.046 [0.120 – 9.152] |
