# Supplementary material for: Short-term Outcomes of Polydioxanone Synthetic Mesh in 2-stage Breast Reconstruction: A Single-Center Comparative Study
Source: Aesthet Surg J. 2026 Jan 24;46(10):1101–8. doi: 10.1093/asj/sjag019 (PMC13577730; doi:10.1093/asj/sjag019)
Supplement: Supplementary file 1 [file sjag019_supplementary_data.zip › Supplemental Table 4.docx]

**Supplemental Table 4**. Patient-level and Breast-level Demographic and Clinical Characteristics by Plane of Reconstruction

| Patient level data | Prepectoral (n = 386) | Subpectoral (n = 138) | p-value |
| --- | --- | --- | --- |
| Age, median [min, max] | 51.0 [24, 75] | 51.0 [25, 78] | 0.659 |
| BMI, median [min, max] | 28.6 [18.9, 51.2] | 25.0 [17.8, 40.2] | <0.001 |
| Current smoker, n (%) | 16 (4.1%) | 15 (10.9%) | 0.008 |
| Diabetes, n (%) | 44 (11.4%) | 11 (8.0%) | 0.334 |
| Perioperative chemotherapy, n (%) | 136 (35.2%) | 35 (25.4%) | 0.044 |
| Bilateral reconstruction, n (%) | 292 (75.6%) | 104 (75.4%) | 1.000 |
| Breast level data | Prepectoral (n = 676) | Subpectoral (n = 241) | p-value |
| Ipsilateral radiation, n (%) | 68 (10.1%) | 23 (9.5%) | 0.917 |
