# Supplementary material for: Short-term Outcomes of Polydioxanone Synthetic Mesh in 2-stage Breast Reconstruction: A Single-Center Comparative Study
Source: Aesthet Surg J. 2026 Jan 24;46(10):1101–8. doi: 10.1093/asj/sjag019 (PMC13577730; doi:10.1093/asj/sjag019)
Supplement: Supplementary file 1 [file sjag019_supplementary_data.zip › Supplemental Table 5.docx]

**Supplemental Table 5**. Patient-level and Breast-level Demographics and Clinical Characteristics of Prepectoral TE

| Patient level data | PDO mesh (n = 126) | Biologic Mesh (n = 181) | No Mesh (n = 79) | p-value |
| --- | --- | --- | --- | --- |
| Age, median [min, max]^1^ | 50 [29, 74] | 50 [24, 75] | 51 [24,68] | 0.760 |
| BMI, median [min, max]^1^ | 28.9 [19.1, 47.9] | 27.5 [18.9, 44.6] | 30.8 [20.6, 51.2] | 0.003^1^ |
| Current smoker, n (%) | 3 (2.4%) | 7 (3.9%) | 6 (7.6%) | 0.205 |
| Diabetes, n (%) | 17 (13.5%) | 14 (7.7%) | 13 (16.5%) | 0.084 |
| Perioperative chemotherapy, n (%) | 47 (37.3%) | 58 (32.0%) | 31 (39.2%) | 0.450 |
| Bilateral reconstruction, n (%) | 97 (77.0%) | 133 (73.5%) | 62 (78.5%) | 0.629 |
| Breast level data | PDO mesh (n = 218) | Biologic Mesh (n = 316) | No Mesh (n = 142) | p-value |
| Ipsilateral radiation, n (%) | 19 (8.7%) | 35 (11.1%) | 14 (9.9%) | 0.677 |

^1^Post-hoc analysis in Supplemental Table 6
