# Supplementary material for: Short-term Outcomes of Polydioxanone Synthetic Mesh in 2-stage Breast Reconstruction: A Single-Center Comparative Study
Source: Aesthet Surg J. 2026 Jan 24;46(10):1101–8. doi: 10.1093/asj/sjag019 (PMC13577730; doi:10.1093/asj/sjag019)
Supplement: Supplementary file 1 [file sjag019_supplementary_data.zip › Supplemental Table 6.docx]

**Supplemental Table 6**. Post-hoc Comparisons of Significant Demographic and Clinical Characteristics from Supplemental Table 5^1^

| Variable | Comparison groups | Unadjusted p-value | Adjusted p-value^2^ |
| --- | --- | --- | --- |
| BMI (median) | Biologic (27.5) vs. PDO mesh (28.9) | 0.162 | 0.486 |
|  | Biologic (27.6) vs. None (30.8) | 0.001 | 0.003 |
|  | PDO mesh (27.6) vs. None (30.8) | 0.041 | 0.123 |

^1^Pairwise two-proportion z-test used. ^2^Bonferroni adjustment applied.
