# Supplementary material for: Short-term Outcomes of Polydioxanone Synthetic Mesh in 2-stage Breast Reconstruction: A Single-Center Comparative Study
Source: Aesthet Surg J. 2026 Jan 24;46(10):1101–8. doi: 10.1093/asj/sjag019 (PMC13577730; doi:10.1093/asj/sjag019)
Supplement: Supplementary file 1 [file sjag019_supplementary_data.zip › Supplemental Table 7.docx]

**Supplemental Table 7**. Post-hoc Comparisons of Breast-Level Complications among Prepectoral TE from Table 4^1^

| Variable | Comparison groups (%) | p-value | Adjusted p-value^2^ |
| --- | --- | --- | --- |
| Wound dehiscence (%) | Biologic (4.4%) vs. PDO mesh (8.3%) | 0.094 | 0.280 |
|  | Biologic (4.4%) vs. None (1.4%) | 0.166 | 0.498 |
|  | PDO mesh (8.3%) vs. None (1.4%) | 0.004 | 0.013 |

^1^Pairwise Fisher’s exact and Chi-squared tests used. ^2^Bonferroni adjustment applied.
