# Supplementary material for: Short-term Outcomes of Polydioxanone Synthetic Mesh in 2-stage Breast Reconstruction: A Single-Center Comparative Study
Source: Aesthet Surg J. 2026 Jan 24;46(10):1101–8. doi: 10.1093/asj/sjag019 (PMC13577730; doi:10.1093/asj/sjag019)
Supplement: Supplementary file 1 [file sjag019_supplementary_data.zip › Supplemental Table 8.docx]

**Supplemental Table 8**. Univariate Regression Analysis of Preoperative and Intraoperative Risk Factors for TE Infection Among Prepectoral TE

|  | p-value | OR [95% CI] |
| --- | --- | --- |
| Age | 0.757 | 1.012 [0.939 – 1.090] |
| BMI | 0.656 | 1.049 [0.851 – 1.292] |
| Ipsilateral radiation therapy | 0.043 | 5.129 [1.042 – 25.231] |
| Perioperative chemotherapy | 0.777 | 1.276 [0.236 – 6.897] |
| Diabetes | 0.860 | 1.248 [0.107 – 14.564] |
| Current smoker | 0.564 | 2.520 [0.109 – 58.344] |
| PDO mesh | 0.600 | 1.628 [0.263 – 10.057] |
| No mesh | 0.966 | 0.951 [0.095 – 9.541] |
