# Supplementary material for: Short-term Outcomes of Polydioxanone Synthetic Mesh in 2-stage Breast Reconstruction: A Single-Center Comparative Study
Source: Aesthet Surg J. 2026 Jan 24;46(10):1101–8. doi: 10.1093/asj/sjag019 (PMC13577730; doi:10.1093/asj/sjag019)
Supplement: Supplementary file 1 [file sjag019_supplementary_data.zip › Supplemental Table 9.docx]

**Supplemental Table 9**. Breast-level Culture Data from Tissue Expander Removals

|  | PDO mesh (n = 38) | Biologic Mesh (n = 57) | No Mesh (n = 25) | p value |
| --- | --- | --- | --- | --- |
| Gram negative culture, n (%) | 7 (18.4%) | 4 (7.0%) | 3 (12.0%) | 0.237 |
| Gram positive culture, n (%) | 13 (34.2%) | 15 (26.3%) | 2 (8.0%) | 0.060 |
| Polymicrobial, n (%) | 1 (2.6%) | 2 (3.5%) | 1 (4.0%) | 1.000 |
| No growth, n (%) | 10 (26.3%) | 18 (31.6%) | 8 (32.0%) | 0.835 |
| No cultures sent, n (%) | 7 (18.4%) | 18 (31.6%) | 11 (44.0%) | 0.090 |
